# Modeling the influence of motor skills on literacy in third grade: Contributions of executive functions and handwriting

**DOI:** 10.1371/journal.pone.0259016

**Published:** 2021-11-29

**Authors:** Margaux Lê, Pauline Quémart, Anna Potocki, Manuel Gimenes, David Chesnet, Eric Lambert

**Affiliations:** 1 Université de Poitiers and, Centre de Recherches sur la Cognition et l’Apprentissage, Poitiers, France; 2 French National Centre for Scientific Research (CNRS), MSHS de Poitiers, Poitiers, France; 3 Laboratoire de Psychologie des Pays de la Loire, Université de Nantes, Nantes, France; 4 Laboratoire de Recherches sur les Apprentissages en Contexte, Université Grenoble Alpes, Grenoble, France; LAPSCO, FRANCE

## Abstract

Several nonlanguage factors influence literacy development, and motor skills are among those most studied. Despite the publication of several studies that have supported the existence of this relationship, the type of influence and underlying mechanisms have been little explored. Herein, we propose modeling the relationship between motor skills and literacy through structural equation modeling, testing the contribution of executive functions and handwriting skills as the possible mediators of this relationship. In a study of 278 third-grade children, we used a wide range of measures related to written language (reading, spelling, reading comprehension, and written production), fine motor skills (dominant hand, nondominant hand, and bimanual dexterity), executive functions (verbal and visuospatial working memory, inhibition, and shifting), and handwriting. Structural equation modeling of the relationship between these different variables indicated that in the third grade, the influence of fine motor skills on literacy is fully mediated by both executive functions and handwriting skills. These motor skills effects are observed for both low levels of processing (reading, spelling) and high levels of processing (reading comprehension, written production). The results are discussed in terms of the potential mechanisms underlying different literacy skills and their implications for pedagogical programs.

## Introduction

The influence of language abilities, such as phonological awareness or rapid automatized naming (RAN), on literacy development has been highlighted in numerous studies (e.g., [1, 2]). However, more and more studies have suggested that nonlanguage abilities also play a role in literacy development [3, 4]. In the current study, we focus on one of these abilities: motor skills. Several arguments from correlational studies (for a review, see [5]) and studies of developmental disorders [6–8] support the hypothesized influence of motor skills on literacy development. However, the mechanisms underlying this link are unclear. Two distinct hypotheses have been suggested to account for this link: mediation by executive functions and mediation by handwriting skills [3, 9]. In the present study, we contrast these two possible mediators of the relationship between motor skills and literacy.

### Relationship between motor skills and literacy

The arguments in favor of the relationship between motor skills and literacy development derive from both studies in the field of developmental disorders and those of typical development. First, in typical development, fine motor skills (FMS) assessed in kindergarten or pre-kindergarten predict reading and spelling levels in higher grades [3, 4, 9–14]. For example, a longitudinal study reported that the manual dexterity of the nondominant hand predicts the level of word reading (*β* = .15) and spelling (*β* = .11) in grade 1, here after controlling for age and phonological skills [10]. Similarly, studies conducted on large samples have reported that visuomotor skills (such as figure copying) assessed in kindergarten explain 10% to 20% of later reading levels (grades 1 to 5) after controlling for early reading skills and socioeconomic status [3, 4, 11–13]. Although most of these studies were conducted in lower grades such as kindergarten, a few studies also showed that motor skills are linked to literacy in older children. For example, Berninger et al. [15] reported that in grades 1 to 3, manual dexterity and figure copying predict the levels of spelling (*β* = .12) and text production (*β* = .12) beyond other predictors. Another study that was conducted with students in grades 3 to 5 showed that the ability to copy a figure still explains 6% of the variance of the literacy measures (text comprehension and production assessment), here again after controlling for demographic factors [16]. All these results indicate that FMS are linked to literacy in typical development (*r* = .16 to .62 depending on the FMS measure; see [5] for a review) and that FMS predict literacy skills beyond already known predictors [3, 4, 9–13]. In addition, studies of children with learning disorders also have supported the existence of a link between reading and motor skills because developmental dyslexia and developmental coordination disorder (DCD) are frequently associated with each other [17–19]. Several studies have shown the existence of motor disorders in children with reading disorders [6–8], and conversely, 70% of children with DCD present an associated reading delay, while only 15% of children without DCD present such difficulties [20].

All these studies have reported a relation between motor skills and literacy development. However, it is necessary to better understand the mechanisms explaining this link. Two hypotheses have been put forward: mediation through executive functions (EFs) [3, 9, 21, 22] and mediation through handwriting [9, 22].

### Hypothesized mediation through executive functions

The first hypothesis attempts to explain the relationship between motor skills and literacy via mediation through EFs. EFs comprise the higher-order cognitive processes that allow for the control and adapting of thoughts and behaviors [23]. Generally, three low-level core EFs are distinguished: *inhibition*, which is the ability to suppress automatic responses or inhibit nonrelevant information; *updating*, which corresponds to the ability to monitor and update working memory; and *shifting* or *cognitive flexibility*, which is the ability to switch between tasks or mental sets [23]. Although some authors have included higher-order EFs, such as planning or problem solving [23], this three-component model appears to be the most commonly used.

Several authors have suggested that EFs could explain the link between motor skills and literacy development [3, 9, 21]. Indeed, the three aforementioned core EFs appear to be the predictors of literacy development [24–27]. For example, Best et al. [24] showed that EFs were related to different measures of literacy (word identification, reading comprehension) in students ranging from 5 to 17 years old (*r* = .27 to .59). Another study reported that different EFs—especially working memory and inhibition—were related to a general measure of reading and writing in kindergarten (*r* = .24 to .51) [26]. Besides their influence on literacy development, EFs are also linked to motor skills. Correlations between FMS and the different core EFs (i.e., working memory, inhibition, shifting) have been identified in kindergarten (*r* = .20 to .60) [28, 29] and in third-grade (*r* = .22 to .37) students [30, 31].

Based on these correlational studies, several authors have suggested that EFs could play a role in the connection between motor skills and literacy [3, 9, 14, 21]. A first explanation suggests that the link between motor skills and literacy can be explained by *general maturation processes*, which are implicated in both motor and cognitive development [9, 14, 22, 30]. In line with this hypothesis, using structural equation modeling (SEM), Michel et al. [14] showed that motor skills assessed in kindergarten predict reading and spelling above IQ and age. However, this influence was not significant after the addition of EFs into the model. According to Michel et al. [14], this result supports a noncausal link between motor skills and literacy that is explained by shared processes between EFs and motor skills. A second explanation suggests that the practice of motor activities can enhance the development of EFs, which, in turn, could play a role in academic achievement [21, 32]. This explanation implies a causal influence of motor skills on EFs and indirectly on literacy development. In line with this hypothesis, several longitudinal studies have used SEM to show that motor skills predict later EFs [16, 21, 32] and that in turn, EFs predict academic success [21, 32–34]. Such mediation was reported in both kindergarten-aged children [32, 34] and in older children from 5 to 10 years of age [21, 33]. However, these studies used measures of global academic achievement, here as assessed through combined literacy and mathematics assessments, but never with specific literacy measures [21, 32–34]. For a thorough understanding of this mediation, it must now be tested with reading and writing measures rather than global academic achievement assessment. Moreover, many of these studies have investigated these relationships in young children—in first grade and below—but a more global vision of this mediation also implies testing students who are more advanced regarding the curriculum they are learning; this would allow to evaluate more diversified measures of literacy, such as text comprehension, that cannot be assessed in younger children. In the current study, we test the extent to which EFs mediate the link between FMS and the development of specific measures of literacy in third-grade students.

### Hypothesized mediation through handwriting

A second hypothesis explains that the link between FMS and literacy is mediated through handwriting, which corresponds to the specific ability to trace letters [35]. This ability needs to be dissociated from FMS, which refer to more general abilities permitting the manipulation of small objects with small hand/finger movements (e.g., peg moving) [9, 12, 13, 35]. Although FMS are important for developing handwriting, the latter ability requires additional language and cognitive skills to produce letters [35]. The mediation hypothesis suggests that better FMS improve the ability to use a pen and enhance handwriting automatization, which, in turn, may facilitate literacy [8, 9, 33, 35]. Several arguments support this hypothesis. First, the development of handwriting as a motor activity is predicted by motor skills, such as manual dexterity [36], visuomotor control [37, 38], and visuospatial integration [37–39]. For example, both manual dexterity and the ability to copy a figure predict writing speed in fifth graders with or without dysgraphia [39]. Visuomotor skills are correlated with the dynamic aspects of handwriting (e.g., velocity or pause duration) in kindergarten (*r* = .55 to .62) [37] and with handwriting quality in second grade (*r* = .25 to .36) [38]. Although these studies focus on young children, FMS probably plays a role later because it is only around 9–10 years of age that handwriting becomes automatic [40, 41]. Second, handwriting skills are a predictor of distinct literacy skills, including reading, spelling, and text production [42–46]. To explain this relationship between handwriting and literacy development, several authors have suggested that the automation of handwriting processing permits the release of cognitive resources during writing, allowing more attention to be given to linguistic processes [35, 47]. In line with this proposal, student performance on the alphabet task, which evaluates handwriting automation, is related to their level in spelling and text production; this link has been reported not only in first grade (*r* = .44-.46) [46], but also in older children through middle school (spelling: *r* = .20-.29; text production: *r* = .21-.62) [42, 43]. Furthermore, handwriting interventions improve spelling and text production from grades 1 to 9 (for a review, see [48]).

Importantly, the studies that have dissociated FMS and handwriting have reported that the effect of FMS on literacy development was not significant when handwriting was controlled for. For example, two longitudinal studies, each conducted with a large sample of kindergarteners, distinguished these two skills and assessed FMS (e.g., peg moving) and handwriting skills (e.g., figure and letter copy) [12, 13]. These studies reported that after controlling for other factors, including linguistic and cognitive skills, only handwriting abilities, and not FMS, predicted literacy acquisition in higher grades (2 to 5). These results support the hypothesized mediation of the link between FMS and literacy through handwriting. However, to the best of our knowledge, this hypothesis has never been tested directly using methods such as SEM, which permits the modeling of mediation effects.

### Two mediations: Exclusive or complementary?

The abovementioned studies suggest that EFs and handwriting mediate the relationship between FMS and literacy. However, previous studies have investigated mediation through one factor or the other, but these two mediations have never been tested simultaneously through SEM to examine their distinct role. Confronting the two mediations is indeed necessary to better understand the mechanisms underlying the motor–literacy association because each mediation may bring something specific to this connection. The two potential mediators could indeed contribute separately to literacy development. For example, hierarchical regression analyses have suggested that both handwriting and EFs contribute independently to text quality in second grade [49] and in text length in fourth grade [50]. The complementarity of these two mediators on literacy has only been shown for text production. However, this result comforts the hypothesis that motor skills influence reading and writing through different pathways.

Additionally, the mediators explaining the link between motor skills and literacy could be different depending on the evaluated literacy dimension. To date, the effect of motor skills on literacy have been tested using nonspecific measures of academic achievement (e.g., [21, 32]) or reading measures only (e.g., [12, 13]). However, written language includes different skills—reading and writing abilities, low-level skills (e.g., word reading, spelling) and high-level skills (e.g., text comprehension, text redaction)—and the various potential mediators could have specific effects on the different literacy dimensions. For instance, the influence of EFs has been identified for different dimensions of literacy, but it seems that they especially play a role in higher levels of written language processing—text comprehension and text production—that require active reasoning about the content. Numerous results have highlighted the effect of EFs on text comprehension (for a review, see [51, 52]) and on both the length and quality of text production [49, 50, 53–55]. On the other hand, handwriting skills may be more related to the dimensions of literacy concerning the learning of writing (spelling and composing). For example, the alphabet task has been linked not only to word spelling [42, 44, 46], but also to higher-level literacy skills, such as the quality of text production [42–44, 49, 50]. Furthermore, some studies have also reported links between handwriting and word reading [13, 35], and here, handwriting interventions seem to improve not only writing but also decoding in kindergarten [56]. Therefore, the use of different measures of literacy can provide a better understanding of the two types of mediations proposed in the literature.

### The present study

A growing number of studies have shown that motor skills influence literacy development throughout different grades [3, 4, 9–14]. Two hypothesized mediations through EF and through handwriting have been suggested and are supported by validated arguments [22, 35]. In the current study, these two different accounts and their exclusivity/complementarity in the explication of the motor–literacy association will be examined. To test these mediation hypotheses, third graders were assessed on measures of FMS (manual dexterity), EFs (inhibition, shifting and working memory), and handwriting (alphabet writing task). These measures were related to their performance on tests of literacy (word reading, word spelling, text comprehension, and quality of text production). Three explanatory models of the relationship between FMS and literacy were compared: mediation through EFs alone, mediation through handwriting alone, and the double mediation of EFs and handwriting.

The choice of the third-grade level is justified by two arguments. First, most studies have been conducted with students in the early stages of literacy development (late kindergarten and early elementary school). Therefore, it is important to examine whether this link is still observed in more advanced grade levels when students begin to automatize handwriting [40, 41]. Moreover, the choice to test the different dimensions of literacy implies that the children have a certain level of expertise with high-level written language (text comprehension and production); as such, the third year of primary school appears to be the lowest level that can be tested to examine the four dimensions of literacy in a single study.

Based on previous research, we predict that FMS will be related to the four dimensions of literacy. We also predict that this association might be explained by a mediation through EFs and by a mediation by handwriting. More precisely, we hypothesize the complementarity of the two mediations, which would depend on the dimensions of literacy tested. We assume that EF mediation would mainly explain the effect of motor skills on high-level literacy skills (e.g., text comprehension and quality of text production), which have often been linked to EFs [49, 51]. We also make the assumption that handwriting mediates the link between motor skills and writing (spelling and quality of text production and spelling) [43, 46], but here, a mediation through handwriting on word reading is also possible [35].

## Materials and methods

### Participants

Two hundred and seventy-eight (278) third graders participated in the current study (mean age = 8;5 years; *SD* = 4 months; 154 girls); they were recruited in 25 classrooms across 16 schools in the Nouvelle Aquitaine region in France. Prior informed parental consent was obtained for each participant. The Ethics Committee for Non-Interventional Studies of the University of Poitiers and Tours provided ethical approval in accordance with the Helsinki Declaration. Before the analyses, children with low IQs and with a profile suggesting developmental dyslexia and/or DCD were excluded. Children with a nonverbal IQ less than 70 (Weschler Nonverbal Scale of Ability [56]), reading difficulties (standardized reading scores lower than 1.5 *SD* on the Batterie Analytique du langage écrit (BALE) reading test [57]) associated with phonological difficulties (standardized phonological awareness or RAN scores lower than 1.5 *SD* on the BALE reading test [57]), or motor difficulties (standardized manual dexterity scores lower than 1.5 *SD* on the M-ABC [58]) were excluded. Hence, the analyses were performed with 250 students (mean age = 8;5 years; *SD* = 4 months; 143 girls).

### Materials

#### Literacy

*Word reading*. To test the participants’ reading skills, a word and pseudo-word reading task from the BALE battery [57] was administered; this is a very comprehensive battery that provides a fine-grained examination of the various dimensions of literacy in French-speaking children from grades 2 to 5. In the reading test, the child was asked to read three lists of 20 items as accurately and as quickly as possible. The three lists were composed of regular words, irregular words, and pseudo-words. Three scores corresponding to the number of correct responses for each list were calculated. The three reading times in seconds were also recorded.

*Word spelling*. The spelling task from the BALE battery [57] was used to assess spelling skills. During this task, the child had to write under dictation three lists composed of 10 regular words, 10 irregular words, and 10 pseudo-words. Three scores corresponding to the number of correctly spelled items in each condition were calculated.

*Text comprehension*. There is no standardized test of text comprehension dedicated to elementary school children in the French language. A text comprehension task developed by Potocki et al. [59] and that has already been used in several studies [60, 61] was administered to evaluate children’s text comprehension skills. In this test, the children were asked to answer 12 literal and inferential questions after reading a short text. They had to choose the good answer among three possible answers. The total score of the correct responses was used as an observed variable.

*Quality of text production*. The writing expression subtest of the Wechsler Objective Language Dimensions (WOLD [62]) was translated into French because no such test already exists in French. It was used to assess the children’s narrative abilities. The instructions comprised the following: “Describe your ideal house with all possible details. For example, you can explain what you prefer in your house and why.” Two different raters assessed the quality of text production by means of two indices: syntax quality (scores from 0 to 5) and vocabulary quality (scores from 0 to 5). The mean of the two scores was then used as a variable for syntax quality and vocabulary quality. In the case of an important disagreement between the two raters (i.e., a difference of more than two points), a third assessment was requested from a third rater. The quality of the text production score (from 0 to 10) corresponded to the aggregate of the scores of the two indices (syntax quality and vocabulary quality).

#### Fine motor skills

To assess FMS, the manual dexterity subtest from the M-ABC [58] was administered. The participants performed three tasks evaluating unimanual dexterity (placing pegs) and bimanual coordination (threading lace). In the manual dexterity task, the child had to place 12 pegs on a board as quickly as possible, first with their dominant hand and then with their nondominant hand. The bimanual coordination consisted of threading a lace in a plank as quickly as possible. Three duration scores (dominant hand dexterity, nondominant hand dexterity, and bimanual coordination) were used as the observed variables.

#### Handwriting

To assess handwriting skills, the children performed the alphabet writing task [15]. This test is one of the most frequently used to assess the level of automatization of handwriting [63, 64]. The children were asked to write the alphabet as quickly as possible within 30 seconds. The number of letters correctly written in 30 seconds was used for the analyses.

#### Executive functions

*Inhibition*. The inhibition subtest of the NEPSY-II [65] was used to evaluate the participants’ inhibition skills. In this task, the experimenter presented the child with a series of forms in black or white. During the first part, the “naming” condition, the child was asked to name the form (square or circle). In the “inhibition” condition, the child had to inhibit their automatic answer and give the opposite answer. In the “switching” condition, the child had to change their answers, depending on the color of the picture. Two variables were extracted: the difference between the time of the “naming” condition and “inhibition” condition (inhibition a) and the difference between the time of the “naming” condition and “switching” condition (inhibition b).

*Shifting*. Two tasks were used to assess shifting abilities: the Trail Making Test [66] and the creature counting item from the TEA-Ch [67]. During the Trail Making Test, the child was asked to link different numbers from 1 to 25 (condition A). Then, the child had to link numbers from 1 to 13 and letters from A to L, alternating numbers and letters (condition B). For the analyses, we used the difference between the execution times of the two conditions (shifting a). In the creature counting item from TEA-Ch, the child had to count creatures in direct and reverse order. The score, which was calculated using the execution time of an item and the number of direction changes, was used for the analyses (shifting b).

*Working memory*. Both verbal and visuospatial working memory were assessed. The letter-number sequencing item of the WISC-V [68] was used to assess phonological working memory. In this task, the children were asked to listen to a list of letters and numbers and provide the numbers in chronological order and then the letter in alphabetical order. To evaluate the visuospatial working memory, the spatial span item of the Wechsler Nonverbal Scale of Ability [56] was administered. In this task, the children had to recall the sequence of cubes touched by the experimenter in the same and reverse order. The dependent variable was the total score, including the performances on the direct and reverse orders.

### Procedure

Trained researchers administered different tests in schools to evaluate motor skills, literacy skills, EFs, and handwriting skills. Phonological skills and IQ were also assessed to exclude children with learning difficulties, but these measures were not included in the analyses. The tasks were distributed in three sessions—one collective session and two individual sessions. The collective session lasted one hour, was administered to the whole class, and included three tasks: the spelling task, the text comprehension task, and the text production task. Then, each child performed two individual sessions of 30 to 45 minutes, alternating between motor tasks, literacy tasks, and cognitive tasks. In the first session, the child performed four tasks: the unimanual dexterity task “placing pegs,” the word reading task, the verbal working memory task, and the Trail Making Test. In the second session, the child performed the creature counting item, the bimanual coordination task “threading lace,” the phonological awareness task (phonological skills), the inhibition task, the RAN task (phonological skills), the matrices (IQ measures), the Corsi blocks, and the alphabet task. The order of the tests in each session was fixed, but the order of the two individual sessions was counterbalanced.

### Statistical analyses

Before the analyses, the outliers—which are defined as the data point below or above three *SDs* from the mean—were removed. Histograms and QQ-plots were examined to assess normality [69]. When the assumption was not checked, transformations were used to normalize the data. Log-transformations—or root-transformation in the case of the presence of 0 values—were used to transform two variables: bimanual coordination and shifting (a). Square transformation was used to normalize six variables: regular word and pseudo-word reading, regular word and pseudo-word spelling, text comprehension, and verbal working memory.

First, correlations between the different variables were assessed. A Benjamini and Hochberg correction was applied to account for increased Type I errors from the multiple analyses [70]. Then, to investigate the relationships between our variables, a confirmatory factor analysis (CFA) and SEM using the Lavaan R package version 3.4.4 were conducted. To assess how well the model fits the data, different indices were examined: chi-square, comparative fit index (CFI), root mean square error of approximation (RMSEA), and standardized root mean square (SRMR). Because the chi-square is sensitive to sample size and model size [71], we decided to accept the model, even if the statistic was below the cut-off (*p* > .05). A CFI value larger than .90, an SRMR value less than .09, and an RMSEA value less than .06 represented a good fit [72]. Different models of mediation were initially compared to confirm the necessity to consider the two mediations (via EFs and handwriting). Then, the best model was used to depict the indirect and direct effects of motor skills on the different literacy latent variables.

## Results

The descriptive statistics for the different variables are reported in Table 1. The analysis of the standardized tests shows that the participants achieved scores in accordance with their age.

**Table 1:** Descriptive statistics for all observed measures.

Measure	*Mean*	*SD*	Min.	Max.
**Literacy skills (scores)**				
Regular word reading accuracy (score/20)	17.4	2.3	10	20
Irregular word reading accuracy (score/20)	9.9	4.3	1	20
Pseudo-word reading accuracy (score/20)	15.3	2.8	7	20
Regular word reading speed (s)	36.4	12.9	13	77
Irregular word reading speed (s)	41.6	15.5	6	90
Pseudo-word reading speed (s)	39.2	11.1	17	78
Regular word spelling (score/10)	6.8	2.4	0	10
Irregular word spelling (score/10)	3.6	2.2	0	10
Pseudo-word spelling (score/10)	7.2	2.04	1	10
Text comprehension (score/12)	8.9	2.4	2	12
Quality of text production (score/10)	5.3	1.2	2	8
**Fine motor skills (time in s)**				
Dexterity (dominant hand)	22.5	3.2	16	31
Dexterity (nondominant hand)	25.8	3.7	18	36
Bimanual coordination	29.4	8.6	16	60
**Executive functions**				
Verbal Working Memory (score)	14.3	3.6	3	20
Visuospatial Working Memory (score)	11.9	2.7	4	18
Inhibition (a) (difference of time in s)	10.3	7.2	-3	46
Inhibition (b) (difference of time in s)	34.4	11.2	-1	78
Shifting (a) (difference of time in s)	95.2	46.7	6	281
Shifting (b) (number of change/time in s)	5.6	1.4	1.2	10.1
**Handwriting**				
Handwriting (number of letters on the alphabet task)	13.0	4.4	4	25

### Correlation analyses

Correlations between literacy measures, motor skills, handwriting, and EFs are shown in Table 2. Significant correlations between literacy scores and motor skills were small (range: .16–.20). The correlations between literacy and the two potential mediators were small to moderate (EFs range: .16–.42; handwriting skills range: .29–.47).

**Table 2:** Correlations between literacy skills (i.e., reading, spelling, text comprehension, quality of text production) and motor skills, executive functions (EFs), and handwriting after Benjamini and Hochberg correction.

	Fine motor skills (FMS)			Executive functions (EFs)						HW
	MD DH	MD NDH	Bim. Coord.	Verbal WM	VS WM	Inhibition (a)	Inhibition (b)	Shifting (a)	Shifting (b)	HW
**Reading skills**										
Regular word reading accuracy	–.11	–.12	–.12	.38***	.11	–.06	–.12	–.05	–.18*	.39***
Irregular word reading accuracy	–.14	–.13	–.16*	.38***	.10	.03	–.06	–.10	–.16*	.47***
Pseudo-word reading accuracy	–.13	–.15	–.08	.26***	.16*	–.04	–.10	–.09	–.20**	.29***
Regular word reading speed	.19*	.10	.04	–.10	.03	.02	–.10	.07	.20**	–.39***
Irregular word reading speed	.19*	.05	–.01	–.09	.01	–.03	.10	.05	.12	–.36***
Pseudo-word reading speed	.20**	.01	–.00	–.10	.00	.05	.12	–.03	.06	–.26**
**Spelling skills**										
Regular words	–.17*	–.18*	–.07	.36***	.20**	–.007	–.06	–.15	–.15	.46***
Irregular words	–.12	–.12	–.13	.22**	.19**	.001	–.05	–.14	–.14	.47***
Pseudo-words	–.03	–.05	–.12	.42***	.12	–.01	–.03	–.08	–.23**	.36***
**Comprehension**										
Text comprehension	–.13	–.15*	–.09	.35***	.22**	–.12	–.04	–.17*	–.09	.29***
**Text production**										
Quality of text production	–.13	–.11	–.11	.34***	.16*	.03	–.08	–.11	–.11	.39***

*Note*. MD: manual dexterity; DH: dominant hand; NDH: nondominant hand; Bim. Coord: bimanual coordination; WM: working memory; VS: visuospatial; HW: handwriting skills; Inhibition (a): NEPSY–item “inhibition”; Inhibition (b): NEPSY–item “switching”; shifting (a): TMT; shifting (b): TEA-Ch.

*p < .05

**p < .01

***p < .001 after Benjamini and Hochberg correction.

### Confirmatory factor analysis

Before testing the mediation of EFs and handwriting, a CFA was conducted with seven latent variables: motor skills, the five literacy variables (reading, spelling, text comprehension, and quality of text production), and the two potential mediators: EFs (verbal working memory, visuospatial working memory, inhibition a, inhibition b, shifting a, and shifting b) and handwriting skills. However, the model did not present a good fit with the data, χ^2^(171) = 410.957, *p* < .001, CFI = .825, RMSEA = .086, SRMR = .094. Therefore, the reading latent variable was divided in two latent variables: reading accuracy and reading speed. The second CFA indicated that the model presented a good fit with the data, χ^2^(164) = 255.531, *p* < .001, CFI = .933, RMSEA = .054; SRMR = .058. All the manifest variables significantly represented their respective latent variables (*p* < .05).

### Model comparison

**Fig 1:**
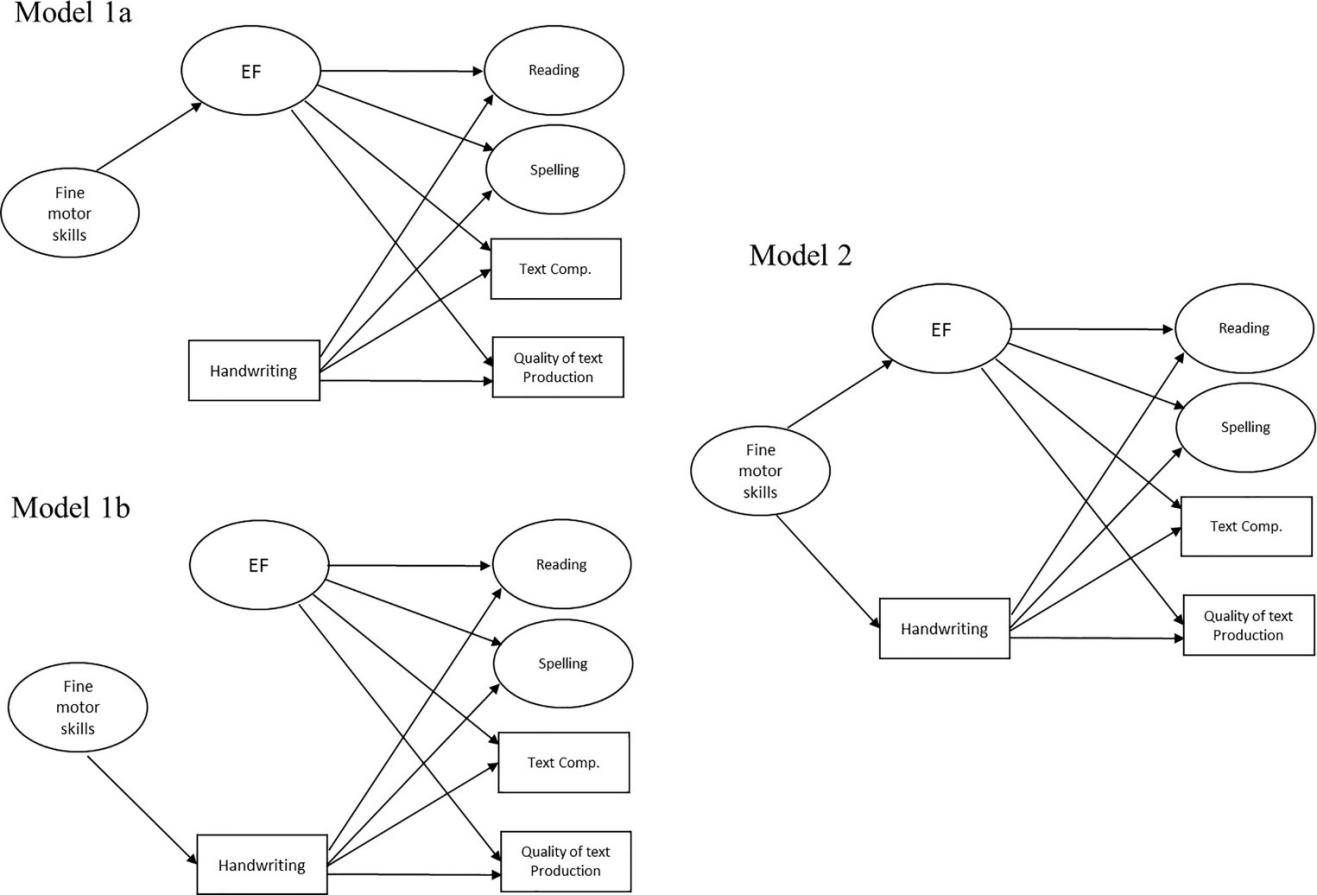
Comparison of the different models to evaluate the mediation model. (1a) Mediation by executive functions alone, (1b) mediation by handwriting alone, and (2) the double mediation model.

The relationships between the variables were then modeled using SEM, including motor skills as the predictor, literacy skills as the outcome, and EFs and handwriting as the two mediators. To investigate the two possible mediations, we compared nested models by adding or suppressing one path at a time [73, 74]. The three models and their fit indices are represented in Fig 1 and Table 3.

**Table 3:** Model fit indices of the three tested models.

	CFI	RMSEA	SRMR
Model 1a	.916	.060	.081
Model 1b	.919	.059	.080
Model 2	.925	.057	.069

We first compared the model with EF mediation alone (Model 1a in Fig 1) to a model adding a path between motor skills and handwriting (Model 2 in Fig 1). The Satorra–Bentler-scaled chi-square difference test indicated that the addition of this path significantly improved the model (Satorra–Bentler Δ*χ*^2^ = 13.28, Δ*df* = 1, *p* < .001). Similarly, we compared the model with handwriting mediation alone (Model 1b in Fig 1) to the double mediation model by adding a path between motor skills and EFs (Model 2 in Fig 1). The Satorra–Bentler-scaled chi-square difference test also indicated that the addition of this path significantly increased the model (Satorra–Bentler Δ*χ*^2^ = 9.32, Δ*df* = 1, *p* = .002). Hence, the model featuring double mediation was retained because it best fit with the data.

### Mediation analysis

**Fig 2:**
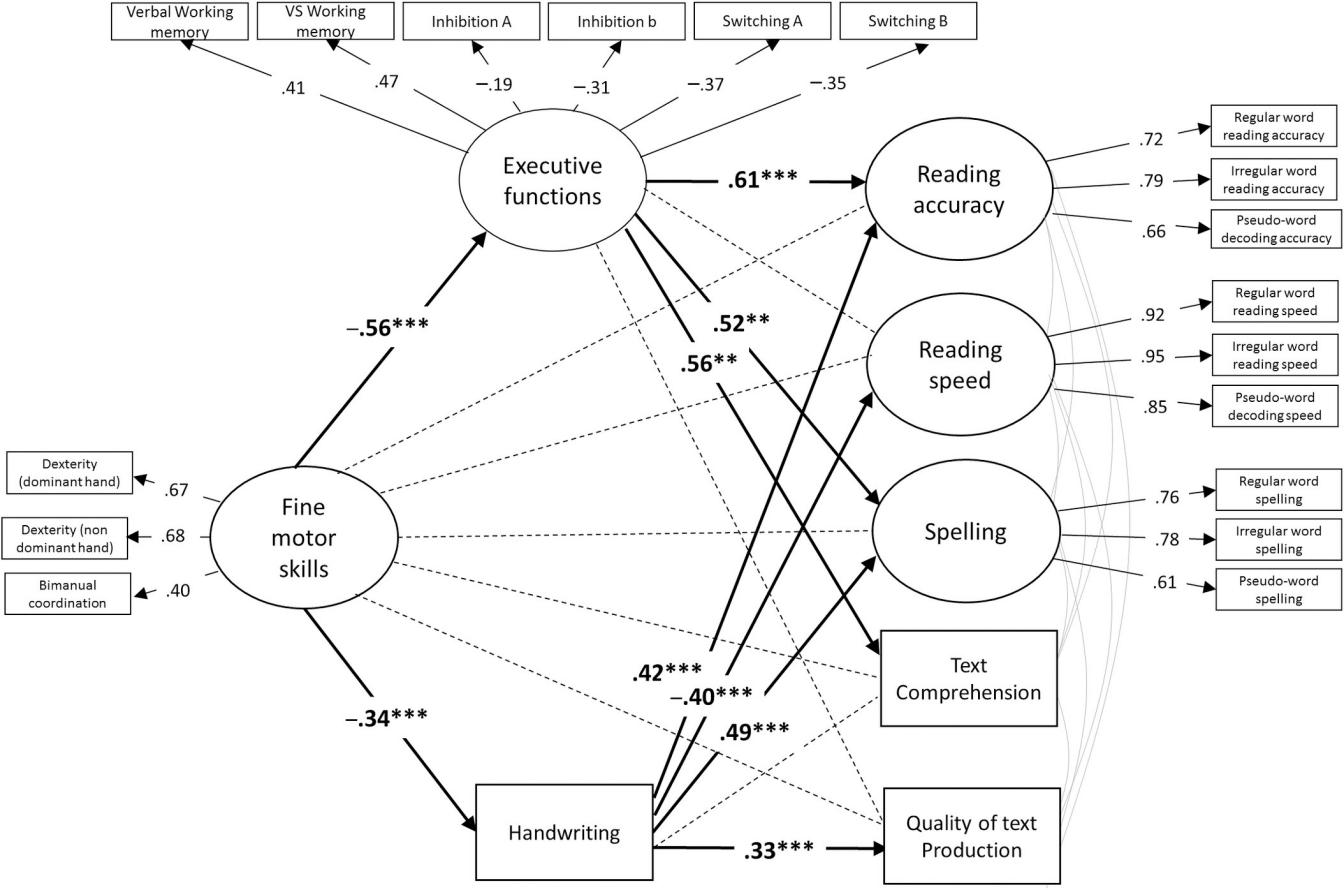
Final mediation model. The model includes motor skills as predictors, executive functions and handwriting as mediators, and the different components of literacy (reading accuracy, reading speed, spelling, text comprehension, and quality of text production) as the outcome variables. Note: *p < .05; **p < .01; ***p < .001.

Finally, we tested the direct and indirect effects of motor skills on the five dimensions of literacy. Because the best model was Model 2, two indirect effects of EF and handwriting were modelized. The model is represented in Fig 2.

The fit indices were good, *χ*^2^(165) = 266.43, *p* < .001, CFI = .926, RMSEA = .057, SRMR = .065. The analyses revealed that the effects of motor skills on literacy were totally mediated by both EFs and handwriting because the direct effects of motor skills on all the literacy dimensions were not significant (reading accuracy: *β* = .25, *p* = .14; reading speed: *β* = .06, *p* = .63; spelling: *β* = .17, *p* = .27; comprehension: *β* = .21, *p* = .18; quality of text production: *β* = .11, *p* = .43). However, the factors that mediate the link between motor skills and literacy depend on the dimensions of literacy. For reading *accuracy*, the indirect effects via handwriting (*β* = -.14, *p* = .004) and EFs (*β* = -.34, *p* = .02) were both significant. Similarly, the results showed that the indirect path through handwriting (*β* = -.17, *p* = .002) and EFs (*β* = -.29; *p* = .02) explains the link between motor skills and spelling. However, the effect of motor skills on reading *speed* was wholly mediated by handwriting (*β* = -.14, *p* = .003), but the path through EFs was not significant (*β* = .009, *p* = .91). A similar result was found for the quality of text production because only handwriting (*β* = -.11, *p* = .005) but not EFs (*β* = -.16, *p* = .08) can explain the effect of motor skills. Conversely, EFs mediated the link between text comprehension and motor skills (*β* = -.31, *p* = .02), but the indirect effect via handwriting (*β* = -.04, *p* = .13) was not significant.

## Discussion

The present study aimed to confirm and explain the mechanisms underlying the link between motor skills and literacy in third-grade students. This relationship has been evidenced across a wide range of studies, especially in young children [5, 22], but the foundations of this link remain to be determined. Two hypotheses have been proposed to explain this relationship, both of which are based on the idea that this link is anchored in mediation by another cognitive function: mediation by EFs [21, 32] and mediation by handwriting skills [13, 35]. These two hypotheses have nonetheless never been contrasted. Therefore, in the present study, we used SEM to examine within the same model the influence of the two mediations proposed in the literature and to investigate their exclusivity/complementarity in explaining the relationship between motor skills and literacy in third-grade students. We assumed that these two cognitive functions both mediate the link between motor skills and literacy in the third grade but that the influence of each of these cognitive functions should depend on the concerned dimension of literacy.

### Executive functions and handwriting: Two complementary mediators

The present study showed that motor skills are linked to different literacy skills in Grade 3. Correlation analyses reported several significant links between motor measures (manual dexterity, bimanual coordination) and literacy measures (reading, spelling and text comprehension. Although the correlation coefficients were small (range: .16–.20), they were similar to those found in previous studies (see [5] for a review). Furthermore, the SEM analysis indicated that motor skills could significantly predict the five dimensions of literacy (i.e., reading accuracy, reading speed, spelling, text comprehension, and text production) through different pathways. The comparison of different models helps in clarifying the distinct influence of handwriting and EFs to explain the motor–literacy relationship. Model comparisons revealed that the one integrating the two mediators (EFs and handwriting) presented the best fit with the data. Previous studies have investigated these mediators in kindergarten and first grade (e.g., [13, 32]), but our study has found that these two factors still underlie the link between motor skills and literacy in third grade, hence extending this result to older children. Furthermore, in accordance with our hypotheses, the results support the importance of considering both mediations; this suggests that different mechanisms explain the influence of motor skills on literacy.

First, our results indicate that EFs partly underscore the motor–literacy relationship. The correlation analyses confirm the link between motor skills and EF development, as has been reported in previous studies [3, 29, 30]. In addition, EFs, especially working memory and flexibility, are linked to different reading and writing skills. The final SEM analysis revealed an indirect effect of motor skills through EFs on the different dimensions of literacy. Our results confirm the mediation already shown in previous studies using general academic measures that combine literacy and mathematics assessments [21, 32], here extending these previous results in a more specific area, that is, literacy. The hypothesis of EFs as a mediating factor in the relationship between motor skills and academic success had already been the subject of investigations [3, 14, 21, 32]. One underlying idea was that the effect of motor skills can be explained, at least partially, by the fact that motor performances actually reflect brain maturation, which affects cognitive functioning and, therefore, academic achievement [14, 22, 33, 75]. Another explanation defends a causal link between motor skills and EFs development. According to this approach, the realization of motor activities is implicated in EF development—possibly because it activates a similar cerebral network [21, 32, 76]. Although the present study is correlational and does not permit resolution to this debate, it provides the first arguments in support of the influence of FMS on EFs and, indirectly, on literacy development.

One benefit of our study was the modeling of an alternative hypothesis to explain the motor–literacy link: the potential mediation through handwriting. According to several authors, better FMS should enhance handwriting acquisition and automatization, which would influence literacy development [35]. Because this hypothesis has been supported by the results of several studies using correlations or regressions (e.g., [13]), none of them have modeled this mediation effect using SEM. Hence, the present study has extended the previous results by investigating this mediation using SEM and confirming that the effect of motor skills on different dimensions of literacy is partly underscored by handwriting skills. Our results have confirmed that motor skills are still related to handwriting skills in third grade; this link was reported in lower grades from kindergarten to second grade [37, 38], but one study reported this link in higher grades as well [39]. Our results show that FMS continue to contribute to third graders’ handwriting skills, even though this age group is characterized by beginning automatization of the writing gesture. However, because the relationship between motor skills and handwriting decreases with age [77], it would be interesting to investigate the mediation through handwriting in older children with more automatized handwriting.

Our results provide a better understanding of the mechanisms that link motor skills to literacy in third graders, here emphasizing the need to consider both the mediators (EFs and handwriting) that explain the motor–literacy relationship in an independent and complementary way. However, several questions remain to be clarified in future research. First, it is possible that a more general cognitive factor may explain the relationships between motor skills and mediator factors. For example, most of the motor, EFs and handwriting tasks used in the current study involved a speed component. Therefore, speed processing could explain a part of the relationships found between these three constructs.

### Effects of executive functions and handwriting depend on the literacy dimensions

One of the strengths of the present study was exploring the relationships between motor skills and low-level literacy skills (reading and spelling), as well as higher-level literacy skills (text comprehension and quality of text production). Until now, no study has investigated the effect of motor skills on different literacy skills. However, depending on the assessed literacy dimension, motor skills and the two potential mediators suggested in the literature (i.e., EFs and handwriting) can influence written language differently. Hence, based on several studies [49–55], we postulated that EFs would mediate the link between motor skills to higher-level skills (text comprehension and text production), whereas handwriting would explain the link between motor and writing skills (word spelling and text production) and word reading. Our findings confirm that idea because the link between motor skills and the different literacy skills is not underlined by the same mediating factors. The results for each literacy dimension and their implications are discussed in detail below.

Concerning *low-level literacy skills*, we hypothesized that the influence of motor skills would be underscored by handwriting but not by EFs. In accordance with our hypothesis, the SEM analysis reported a handwriting mediation for both word reading and spelling; this finding is in line with abundant results that links handwriting and spelling [15, 46, 48], which was mainly interpreted as a release of the cognitive resources with the automation of handwriting that permit greater allocation to orthographic processing during writing tasks [35]. Concerning word reading, previous findings have suggested that developing lexical representations for words through handwriting provides additional motor information that contributes to the memorization of new letters and words in the lexicon [78]. Handwriting may permit the construction of a sensorimotor representation of the word that could be reactivated during both writing and reading [79]. It has been shown that learning through handwriting compared with reading alone improves the acquisition of new letters and words [37, 78, 80–82]. For example, in fifth graders, it has been found that the recall of a new orthography is enhanced if the word is copied (handwriting condition) rather than spelled aloud during the learning phase [78]. Furthermore, in kindergarten, letter recognition is improved after learning by handwriting compared with learning by tapping in adults and in preschool children [80]. Conversely, the artificial alteration of graphomotor movement during learning by copy decreases letter and pseudo-word recognition in kindergarteners [35]. Our results are consistent with these studies, thus showing that the link between FMS and reading/spelling level is still mediated by handwriting level in third grade. This suggests that the automatization of the graphomotor gesture is still crucial in the development of literacy in this age group. However, another explanation could also be provided to explain the link between handwriting and reading/spelling. One of the inherent characteristics of this handwriting task (the alphabet task) is that it involves a language component (i.e., letter knowledge). Therefore, this language dimension shared by handwriting and literacy may explain this mediation.

Second, our results reveal that EFs also mediate the link between FMS and two low-level literacy skills: reading accuracy and spelling. This mediation was not predicted from our literature review. However, the model incorporates a measure of phonological working memory that could explain the relationship between EFs and low literacy skills. The implication of phonological working memory in reading and spelling acquisition has indeed been extensively investigated, and this EF is a strong predictor of literacy acquisition, regardless of the grade level [83]. In addition, other results on nonverbal EFs are consistent with our findings because they have reported that inhibition and shifting are also linked to reading and spelling in third grade [84]. Finally, two measures of reading (i.e., reading accuracy and reading speed) were incorporated in the model, but the SEM analysis showed that these two components were distinct and not related to the same factors. Although reading accuracy is related to both mediators, reading speed is related only to handwriting. This is quite surprising given that word reading efficiency has been linked to inhibition and shifting [84]. Further studies are needed to better understand these results. To summarize, the reading and spelling level of third-grade students is related to their FMS, and this relationship is explained by their handwriting and EFs skills, which mediate this relationship.

Concerning high-level skills, we hypothesized that EFs mediate the effect of motor skills for both text comprehension and text production. Moreover, we expected that handwriting mediates the link between motor skills and text production, which is in accordance with previous studies [49, 50, 88].

First, in accordance with our hypotheses, our results on *text comprehension* show that EFs—but not handwriting—mediate the effect of motor skills. Our model is in line with numerous studies that have reported an implication of high cognitive processes in text comprehension in various grade levels [51]. For example, updating and inhibition have been shown to predict text comprehension from 8 to 16 years of age [85–87]. The underlying assumption is that these two EFs facilitate comprehension by maintaining the relevant information and suppressing nonrelevant information during text comprehension [51, 61]. On the contrary, text comprehension is not significantly predicted by the alphabet task, which is not surprising given that comprehension does not imply a graphomotor gesture. As we developed earlier, graphomotor processing during writing tasks could enhance reading development. This mechanism could explain the link between motor skills and word reading in the present study. However, it does not apply to text comprehension in the third grade.

Second, concerning *text production*, we hypothesized that both mediator factors (EFs and handwriting) could predict the measure of text production quality, as has been shown in previous studies focusing on the same age range [50]. In accordance with our hypotheses, the SEM analysis showed that handwriting mediates the link between motor skills and quality of text production. This is consistent with the simple view of writing proposed by Berninger et al. [47], which has suggested that low-level skills such as handwriting and spelling are necessary to succeed in text production. More automatized handwriting may increase the resources that are available for higher levels, such as text planning or revising during writing, because fewer resources would be devoted to motor control. In contrast, our results did not report a significant link between EFs and text production. Although this has been described by previous studies [49, 50, 53, 88], several explanations can be offered to explain these divergent results. The first explanation is that the inclusion of another mediator in the model—handwriting—may have reduced the influence of EFs on composing. However, previous findings have reported that EFs predict text production independently of handwriting [49, 50, 88]. Another explanation could come from the differences in methodology; in our study, the students had to write a descriptive text, while in other studies, a narrative text was written [49, 50, 88]. However, the type of text to be produced influences both the syntax complexity in production (e.g., the number of words by clauses) [89] and strategies employed during the writing process [90]. Therefore, it is possible that the involvement of EFs in writing differs according to the type of text to be produced.

Finally, our results show that the effect of motor skills on the different dimensions of literacy occurs through different pathways: both EFs and handwriting mediate this link for low-level skills (reading accuracy and spelling), but the effect of motor skills can be explained only by EFs for text comprehension and only by handwriting for text composing and reading speed.

To conclude, numerous studies have focused on the language abilities that predict literacy development [1, 2]. The present study has showed that motor skills also play a role in literacy in the third grade and brings new elements to explain this relationship. Two factors underlying the motor–literacy relationship were identified: handwriting and EFs. Their mediation effect was specified as a function of the literacy skills examined. The results highlight the necessity of distinguishing the different literacy components for finely analyzing the underlying processes explaining the motor–literacy relationship and, more generally, the predictors of literacy development, such as EFs and handwriting skills. Furthermore, although the question of causality cannot be answered here, the double mediation with handwriting skills and EFs is a primary argument for the implementation of a combined training program based on these two domains. Numerous previous studies have shown the beneficial effect of phonological abilities training, even for third-grade students [91]. Future research will need to test whether training that involves handwriting tasks and exercises that improve EFs has a positive effect on learning written language. If this hypothesis is validated, then it will strengthen the causal link between mediators (handwriting and EFs) and literacy. Furthermore, this type of training program could be an additional avenue to help students with a specific learning difficulty acquire reading and writing skills.

## Supporting information

S1 File Excel table containing data from all participants. https://figshare.com/articles/dataset/Modeling_the_influence_of_motor_skills_on_literacy_in_third_grade_xlsx/14724447.
(XLSX)
